# Sexuality, fertility and family planning characteristics of married women aged 15 to 19 years in Ethiopia, Nigeria and Tanzania: a comparative analysis of cross-sectional data

**DOI:** 10.1186/s12978-019-0666-0

**Published:** 2019-01-21

**Authors:** Christina J. Atchison, Jenny A. Cresswell, Saidi Kapiga, Mussa Kelvin Nsanya, Emily E. Crawford, Mohammed Mussa, Christian Bottomley, James R. Hargreaves, Aoife M. Doyle

**Affiliations:** 1 London School of Hygiene and Tropical Medicine, MRC Tropical Epidemiology Group, London, UK; 2 Mwanza Intervention Trials Unit, Mwanza, Tanzania; 3 Binomial Optimus Limited, Abuja, Nigeria; 4 MMA Development Consultancy, Addis Ababa, Ethiopia; 5 London School of Hygiene and Tropical Medicine, Department of Social and Environmental Health Research, London, UK

**Keywords:** Adolescents, Contraception, Family planning, Reproductive health, Africa

## Abstract

**Background:**

Adolescents 360 (A360) is an initiative being rolled out across Ethiopia, Nigeria and Tanzania with the aim of increasing uptake of voluntary modern contraception among sexually active women aged 15 to 19 years. Using evaluation baseline survey data, we described key sexuality, fertility and contraceptive use characteristics of married women aged 15 to 19 years living in three sub-national settings.

**Methods:**

Cross-sectional baseline surveys of married women aged 15 to 19 years were conducted in Oromia (Ethiopia), Nasarawa (Northern Nigeria), and Mwanza (Tanzania) between August 2017 and February 2018. We also interviewed the husbands of a sub-group of married respondents to measure spousal acceptance and support for adolescent women to use modern contraception. A clustered sampling design was used in all three countries. We produced descriptive statistics on the socio-demographic and sexual and reproductive health characteristics of married women aged 15 to 19 years by study setting.

**Results:**

In Oromia, Nasarawa and Mwanza, 31.4% (327/1198), 27.4% (1321/4816) and 7.5% (15/201) of married women surveyed had no education, and 68.3, 81.3 and 83.1% had ever been pregnant, respectively. Unmet need for modern contraception was 20.5, 21.9 and 32.0% in married women in Oromia, Nasarawa and Mwanza, made up almost entirely of unmet need for spacing. The vast majority of married women surveyed in Oromia (89.1%) and Mwanza (90.1%) had seen or heard about contraception in the last 12 months, compared to 30.1% of those surveyed in Nasarawa. Modern contraceptive prevalence (mCPR) was highest in married women aged 15 to 19 years in Oromia (47.2%), followed by Mwanza (19.4%) and Nasarawa (8.7%). Of those using a modern method of contraception in Oromia, 93.4% were using injectables or long-acting methods, compared to 49.4% in Nasarawa and 69.6% in Mwanza.

**Conclusions:**

Overall, unmet need for modern contraception is high among married women aged 15 to 19 years across the three settings. mCPR for married women aged 15 to 19 years is low in Nasarawa and Mwanza. Ultimately, no single intervention will suit all situations, but improving the quality, analyses and utilisation of subnational data can help decision-makers design more context specific interventions.

## Plain English summary

Reducing pregnancies among adolescents is a global priority. Adolescents 360 (A360) is a sexual and reproductive health programme being rolled out across Ethiopia, Nigeria and Tanzania to increase use of modern contraception among sexually active women aged 15 to 19 years. To better understand the target population for the programme, in this study, we sought to describe sexuality, fertility and contraceptive use characteristics of married women aged 15 to 19 years living in three settings.

Surveys of married women aged 15 to 19 years were conducted in Oromia (Ethiopia), Nasarawa (Northern Nigeria), and Mwanza (Tanzania). We also asked husbands of female respondents whether they supported adolescent women using modern contraception. The majority of adolescent women reported some level of education, and had been pregnant in the past. In Oromia and Mwanza, most respondents had seen or heard about contraception in the last 12 months compared to only a third in Nasarawa. Unmet need for contraception, defined as the proportion of women wishing to stop or delay having children but who are not using contraception, was high. The use of modern contraception was low but highest in Oromia, followed by Mwanza and Nasarawa. Attitudes of female respondents and their husbands towards contraceptive use were positive and broadly similar with respect to approving of married couples using a modern contraception to avoid or delay pregnancy.

In conclusion, improving the collection and use of local sexual and reproductive health data can help decision-makers tailor interventions to the needs of their local population.

## Background

Reducing pregnancies among adolescents is a global priority [1, 2]. Specifically, increasing contraceptive use in adolescents is a top priority for the international Family Planning 2020 (FP2020) initiative [1]. It is an important aspect of three of the 13 targets found in the United Nations (UN) Sustainable Development Goal (SDG) for health (SDG 3) [3], including by 2030, (1) reducing the global maternal mortality ratio, (2) ending preventable deaths of newborns and children under 5 years of age, and (3) ensuring universal access to sexual and reproductive health-care services [3].

Countries in sub-Saharan Africa have some of the lowest levels of modern contraceptive prevalence (mCPR) globally [4]. Use of modern contraceptives among adolescents, especially married adolescents, in sub-Saharan Africa is particularly low [5]. The factors that contribute to low mCPR among adolescents include early marriage and the desire to demonstrate fertility, lack of comprehensive sexuality education, holding misconceptions about contraception, fear of side effects and infertility, financial cost, policies preventing the provision of contraceptives, and negative societal norms and stigma around contraception [5–7]. In developing countries, 90% of childbearing between the ages of 10 and 19 takes place within the context of child marriage [8], and complications related to childbearing are the leading cause of mortality among adolescent girls aged 15 to 19 worldwide [9]. Child marriage has shown to be associated with unintended pregnancy, low levels of contraceptive use, and limited use of maternal health services, which result in increased vulnerability for negative maternal outcomes [10]. Unmet need for contraception, defined as the proportion of women wishing to limit or postpone child birth but who are not using contraception, quantifies the gap between women’s reproductive intentions and their contraceptive behaviour [11]. On average, sexually active unmarried adolescents experience a higher percentage of unmet need for contraception than those who are married; however, married adolescents experience a higher percentage of unmet need than married women in other age groups [11]. Socio-cultural and structural barriers often prevent adolescents from achieving their reproductive intentions, which can result in unintended pregnancies [11]. New interventions are needed to effectively address these issues [12]. This requires a better understanding of current sexuality, fertility and contraceptive use characteristics among adolescents.

The Adolescents 360 (A360) programme is being rolled out across Ethiopia, Nigeria and Tanzania with the aim of increasing uptake of voluntary modern contraception among sexually active women aged 15 to 19 years [13]. The final A360 intervention is country-specific, and includes a combination of sexual and reproductive health education (through health clinic and community events), livelihood related counselling, and improved contraceptive provision through adolescent friendly services [13]. The external evaluation of A360 comprises of an outcome evaluation, a process evaluation and a cost effectiveness study. As part of the outcome evaluation, baseline survey data was collected on the target population in three sub-national settings. As independent evaluators of the A360 programme, we had no involvement in the selection of countries targeted for the intervention. Therefore, the cross-country comparison presented here is based on availability of baseline survey data from the main outcome evaluation study. However, it is a chance to share findings from three countries with a mix of characteristics, including geography, culture, religion, and contraceptive prevalence representing some of the diversity in sub-Saharan Africa. In addition, although country-specific DHS and FP2020 surveys routinely collect these data in sub-Saharan Africa, the numbers of adolescents in these surveys are small [1, 14–16]. Therefore, this is an opportunity to build a comprehensive picture of sexuality, fertility and contraceptive use characteristics of married women aged 15 to 19 years in these countries.

The aim of this study is to describe sexuality, fertility and contraceptive use characteristics of married women aged 15 to 19 years living in three sub-national settings in Ethiopia, Nigeria and Tanzania.

## Methods

### Study design and settings

Between August 2017 and February 2018, we conducted cross-sectional baseline surveys among married women aged 15 to 19 years in Oromia (Ethiopia), Nasarawa (Northern Nigeria), and Mwanza (Tanzania). The surveys were part of a comprehensive outcome evaluation to assess the impact of the A360 programme on a number of sexual and reproductive health (SRH) outcomes, primarily uptake of voluntary modern contraception among sexually active women aged 15 to 19 years. The full multi-country A360 outcome evaluation study protocol is described elsewhere [17]. Although both married and unmarried women were surveyed in some study regions [17], here we present results for married women only.

In Ethiopia, the programme is being implemented in four regions (Amhara, Oromia, State of Southern Nations, Nationalities and People’s Region (SNNP) and Tigray). The baseline survey in Ethiopia was conducted in four woredas (districts) in Oromia region [17].

In Nigeria, A360 is being implemented in seven southern states (Lagos, Osun, Ogun, Oyo, Edo, Delta and Akwa Ibom) and three northern states (Federal Capital Territory, Nasarawa and Kaduna). The baseline survey of married women aged 15 to 19 years was conducted in four local government areas (LGAs) in Nasarawa State [17].

In Tanzania, A360 is being implemented in 16 regions (Kagera, Geita, Mwanza, Arusha, Dodoma, Tabora, Shinyanga, Simiyu, Tanga, Dar es Salaam, Pwani, Lindi, Mtwara, Mbeya, Iringa, and Morogoro). The baseline survey was conducted in urban and semi-urban wards of Ilemela district, Mwanza region [17].

### Study population

We included in the study women aged 15 to 19 years who were married or living as married, and were living in the study sites at the time of the survey. Only women who voluntarily provided informed consent were interviewed.

To measure spousal acceptance and social support for adolescent women to adopt SRH behaviours, our second target study population were the husbands of surveyed married women. These men were invited to be interviewed after the married woman had granted permission to do so.

### Sampling strategy and sample size

Full details of the sampling strategy and sample size calculations are described elsewhere [17]. A clustered sampling design was used in all three countries. In each country we used the smallest available administrative unit as the primary sampling unit (PSU) and interviewed all eligible women living in the sampled unit. Specifically, we used kebeles from the 2007 census in Oromia (Ethiopia), enumeration areas (EAs) from the 2006 census in Nasarawa (Nigeria), and streets in Ilemela district, Mwanza (Tanzania).

#### Oromia, Ethiopia

A sample of 57 kebeles was selected from across the four study woredas with probability proportional to the kebele population size. Our target sample size for the baseline survey was 1041 married women aged 15 to 19 years and 128 husbands [17].

#### Nasarawa, Nigeria

A simple random sample of 621 EAs was selected across the four LGAs. Our target sample size for the baseline survey was 4600 married women aged 15 to 19 years and 250 husbands [17].

#### Mwanza, Tanzania

A simple random sample of 34 ‘streets’ was selected across the 15 urban and semi-urban wards of Ilemela district. As per study protocol, in the first eight ‘streets’, we randomly selected 50 GPS coordinates using ArcGIS software version 9.3 (Esri, Redlands, USA). All households whose front door was located within a radius of 20 m around the GPS point were visited and all eligible consenting women aged 15 to 19 years residing in these households were invited to be interviewed [17]. Fewer eligible women than predicted were surveyed using this sampling strategy, thus in the remaining 26 ‘streets’ we visited all households and administered the questionnaire to all eligible and consenting women aged 15 to 19 years. Our target sample size for the baseline survey was 193 married women aged 15 to 19 years and 19 husbands [17].

The overall outcome evaluation study was powered to detect changes in mCPR in our target population (married women aged 15 to 19 years). Therefore, due to resource constraints it was possible only to include a small sample of husbands in each setting [17].

### Tool for baseline survey

The questionnaires were adapted from respective country Demographic and Health Survey (DHS) [14–16] and FP2020 survey instruments [1]. Questionnaires were administered face-to-face using tablets by female interviewers aged between 18 and 26 years [17].

The questionnaire had three components: (1) socio-demographic characteristics, (2) fertility characteristics and preferences, and (3) contraceptive knowledge, attitudes and practices. Only married female respondents who reported sexual activity in the last 12 months were considered sexually active and asked about contraceptive use [17].

### Study outcomes

Sexuality and fertility characteristics included: age at first sexual intercourse, timing of last sexual intercourse, current pregnancy status, ever been pregnant, ever given birth, age at first birth, number of living children, planning status of most recent birth, and unmet need for modern contraception (as per DHS definition [18]).

Family planning characteristics included: mCPR, heard about modern contraception and sources of information on contraception, approval of married couples using a modern contraceptive method to avoid or delay pregnancy, knowledge of the benefits of contraception, misconceptions about contraception, and self-efficacy to use modern contraception.

As per DHS definition, this study considered three outcomes for unmet need for modern contraception: total unmet need, unmet need for spacing, and for limiting. The denominator for the calculation of unmet need is the total of currently married women aged 15–19 years [18]. The numerator includes only women who were not using contraception at the time of the survey. The nonusers were first split into pregnant or postpartum amenorrhoeic (menstrual period not returned following a birth during the 2 years preceding the survey) women on one side, and those who were neither pregnant nor postpartum amenorrhoeic on the other. The pregnant or postpartum amenorrhoeic were then classified by whether the pregnancy or last birth was wanted at that time or unwanted. Women in the mistimed or unwanted category were considered having the unmet need for spacing and for limiting respectively [18]. The other component of unmet need is composed of women who were neither pregnant nor postpartum amenorrhoeic. These women were further divided, into fecund and infecund. Fecund women who wanted children two or more years in the future, or were undecided whether/when they wanted a child were regarded as having an unmet need for spacing. Fecund women who wanted no more children were regarded as having an unmet need for limiting [18]. The total unmet need was composed of unmet need for spacing plus the unmet need for limiting.

mCPR among 15 to 19 year old married women was defined as per the DHS definition [16]:

$$ \frac{\mathrm{Number}\ \mathrm{of}\ \mathrm{married}\ 15-19\hbox{-} \mathrm{year}\hbox{-} \mathrm{old}\ \mathrm{women}\ \mathrm{reporting}\ \mathrm{use}\ \mathrm{of}\ \mathrm{modern}\ \mathrm{contraceptives}\ \mathrm{at}\ \mathrm{the}\ \mathrm{time}\ \mathrm{of}\ \mathrm{the}\ \mathrm{survey}}{\mathrm{Number}\ \mathrm{of}\ \mathrm{married}\ 15-19\hbox{-} \mathrm{year}\hbox{-} \mathrm{old}\ \mathrm{women}} $$

Modern contraception was defined to include the following [16]: male and female sterilisation, contraceptive implants, intrauterine contraceptive devices (IUCD), injectables, oral contraceptive pill, emergency contraceptive pill, male condom, female condom, Standard Days Method (SDM), Lactational Amenorrhoea Method (LAM), diaphragm, spermicides, foams and jelly.

Knowledge of the benefits of contraception was assessed through five questions, including whether the woman agreed with the following statements: (1) preventing unwanted pregnancies is a benefit of contraception, (2) some contraception methods reduce sexually transmitted infections, (3) modern contraception can help an adolescent woman delay the birth of her first child, if she wants to, (4) after she begins to have children, modern contraception can allow an adolescent woman to decide when to have another child, and (5) using modern contraception can allow an adolescent woman girl to complete her education, find a better job and have a better life.

Misconceptions about contraception were assessed through four questions, including whether the woman believed that: (1) some modern contraception can stop an adolescent woman from ever being pregnant again even after she stops using it, (2) if a modern contraception changes an adolescent woman’s menstrual bleeding, it’s bad for her health and can harm her womb, (3) some modern contraceptives can make adolescent women permanently fat, and (4) adolescent women who use modern contraception are promiscuous.

Self-efficacy was assessed through four questions relating to the woman’s ability to access and use family planning methods, including whether she: (1) felt able to start a conversation with her partner about contraception, (2) felt able to use a method of contraception even if her partner did not want her to, (3) felt able to obtain information on contraception services and products if she needed to, and (4) felt able to obtain a contraception method if she decided to use one.

### Statistical analysis

All analyses were conducted in Stata 15.

Descriptive statistics on the socio-demographic, and sexual and reproductive health characteristics of married women aged 15 to 19 years were produced for each study region.

We used sampling weights and robust standard errors to account for the clustered sampling design. The unit of clustering was kebele in Oromia, EA in Nasarawa, and street in Mwanza.

## Results

In Oromia, 93.4% (1198/1282) of potentially eligible married women were interviewed. We also interviewed 142 husbands. In Nasarawa, 97.0% (4816/4963) of potentially eligible married women were interviewed. We also interviewed 326 husbands. In Mwanza, where both married and unmarried women were surveyed, 68.6% (3511/5121) of potentially eligible women were interviewed, of which 5.7% (201/3511) were married women aged 15 to 19 years. We also interviewed 16 husbands. The most common reasons for not interviewing a potentially eligible woman in all study settings were being absent or unavailable after a maximum of three visits.

### Socio-demographic characteristics of married women aged 15 to 19 years

In Oromia, the mean age of respondents was 17.8 years (standard deviation (SD) 1.1). The median age at the time of marriage was 16 years (range 11–19 years). About one third of respondents had no education and just over half reported primary education as their highest level of education attained (Table 1). The main religion was Orthodox Christian (65.9%) (Table 1).

**Table 1:** Socio-demographic characteristics of married women aged 15–19 years

Characteristic	*n* (%) ^a^					
	Oromia, Ethiopia *N* = 1198		Nasarawa, Nigeria *N* = 4816		Mwanza, Tanzania *N* = 201	
Age (years): Mean (SD)	17.8 (1.1)		17.6 (1.3)		17.2 (1.2)	
Age at marriage (years)	*n* (%)^a^	Median (IQR)	*n* (%)^a^	Median (IQR)	*n* (%)^a^	Median (IQR)
< 15	167 (12.7)	14 (13–14)	929 (19.3)	14 (13–14)	4 (2.0)	12 (12–12)
15–17	840 (71.3)	16 (15–17)	3172 (65.9)	16 (15–17)	107 (53.2)	17 (16–17)
18–19	191 (16.0)	18 (18–18)	669 (13.9)	18 (18–18)	90 (44.8)	19 (19–19)
Don’t know	0 (0)	–	46 (0.96)	–	0 (0)	–
Overall	1198 (100.0)	16 (15–17)	4816 (100.0)	16 (15–17)	201 (100.0)	17 (16–18)
Age of husband (years)	n (%)^a^	Median (IQR)	n (%)^a^	Median (IQR)	n (%)^a^	Median (IQR)
15–19	10 (7.4)	18 (18–19)	0 (0)	–	0 (0)	–
20–24	71 (49.0)	22 (21–24)	22 (6.5)	22 (21–23)	6 (37.5)	22 (21–23)
25–29	55 (40.5)	26 (25–27)	98 (30.1)	27 (26–28)	7 (43.8)	27 (26–29)
> 30	6 (3.1)	32.5 (32–35)	206 (63.4)	34 (31–37)	3 (18.8)	37 (30–40)
Overall	142 (100.0)	24 (22–26)	326 (100.0)	30 (28–35)	16 (100.0)	26.5 (22.5–29)
Highest level of education						
No education	327 (31.4)		1321 (27.4)		15 (7.5)	
^b^ Quranic	N/A		125 (2.6)		N/A	
Primary	695 (54.7)		1173 (24.4)		131 (65.2)	
Secondary	172 (13.7)		2082 (43.2)		55 (27.4)	
Higher/Technical/Vocational	4 (0.24)		113 (2.3)		0 (0)	
Don’t know	0 (0)		2 (0.04)		0 (0)	
Religion						
Roman Catholic	0 (0)		542 (11.3)		61 (30.4)	
Orthodox Christian	868 (65.9)		0 (0)		0 (0)	
Protestant	106 (7.8)		1941 (40.3)		99 (49.3)	
Muslim	205 (24.7)		2310 (48.0)		38 (18.9)	
Traditional	18 (1.5) (18)		19 (0.39)		0 (0)	
No religion	1 (0.07)		4 (0.08)		3 (1.5)	
Currently do any activity to earn money						
Yes	180 (13.4)		2022 (42.0)		32 (15.9)	
No	1018 (86.6)		2788 (57.9)		169 (84.1)	
No response	0 (0)		6 (0.12)		0 (0)	

^a^ Numbers and percentages may not match exactly because the analysis used sampling weights to account for the sampling design

^b^ In Nigeria, apart from the formal educational system, a non-formal Arabic and Islamic Educational System operates among the Nigerian Muslims, through Quranic schools

In Nasarawa, the mean age of respondents was 17.6 years (SD 1.3). The median age at the time of marriage was 16 years (range 5–19 years). About one third of respondents had no education and 43.2% reported secondary education as their highest level of education attained (Table 1). The main religion was Islam (48.0%) followed by Protestant (40.3%) (Table 1).

In Mwanza, the mean age of respondents was 17.2 years (SD 1.2). The median age at the time of marriage was 17 years (range 13–19 years). Few respondents had no education (7.5%) and 65.2% reported primary education as their highest level of education attained (Table 1). The main religion was Christianity (79.6%) (Table 1).

### Sexuality and fertility characteristics of married women aged 15 to 19 years

In Oromia, 98.9% of married women aged 15 to 19 years had been sexually active during the past 12 months. The mean age of first sexual intercourse was 16.0 years (SD 1.4). A total of 805 (68.3%) married women surveyed had ever been pregnant (Table 2). Overall, 51.2% (610 of 1198) of married women surveyed had given birth. The mean age at first birth was 16.9 years (SD 1.3). Overall, about a quarter of births were reported as mistimed (wanted at a later time).

**Table 2:** Sexuality and fertility characteristics of married women aged 15–19 years

Characteristic	*n* (%) ^a^		
	Oromia, Ethiopia *N* = 1198	Nasarawa, Nigeria *N* = 4816	Mwanza, Tanzania *N* = 201
Age at first sexual intercourse: Mean (SD)	16.0 (1.4)	15.1 (1.7)	16.0 (1.6)
Timing of last intercourse			
Within past 4 weeks	1099 (90.8)	2411 (50.1)	138 (68.7)
Within past year	87 (8.1)	1956 (40.6)	49 (24.4)
More than 1 year	11 (0.98)	350 (7.3)	14 (7.0)
Never had sex	0 (0)	15 (0.31)	0 (0)
Don’t know	0 (0)	16 (0.33)	0 (0)
No response	1 (0.04)	68 (1.4)	0 (0)
Ever been pregnant			
Yes	805 (68.3)	3913 (81.3)	167 (83.1)
No	392 (31.7)	893 (18.5)	34 (16.9)
Don’t know	1 (0.04)	8 (0.17)	0 (0)
No response	0 (0)	2 (0.04)	0 (0)
Currently pregnant			
Yes	200 (17.7)	1504 (31.2)	51 (25.4)
No	983 (81.2)	3181 (66.1)	146 (72.6)
Don’t know	15 (1.2)	125 (2.6)	4 (2.0)
No response	0 (0)	6 (0.12)	0 (0)
Ever given birth			
Yes	610 (51.2)	2650 (55.0)	110 (54.7)
No	588 (48.8)	2166 (45.0)	91 (45.3)
Age at first birth: Mean (SD)	16.9 (1.3)	16.3 (1.6)	17.0 (1.2)
Number of living children			
No children	600 (49.9)	2328 (48.3)	97 (48.3)
1 child	520 (43.5)	1665 (34.6)	85 (42.3)
2 children	74 (6.3)	681 (14.1)	18 (9.0)
3 or more children	4 (0.24)	142 (2.9)	1 (0.50)
Planning status of most recent birth at the time they gave birth	*N* = 610	*N* = 2650	*N* = 110
Wanted then	408 (69.8)	2264 (85.4)	49 (44.5)
Wanted later	173 (26.1)	348 (13.1)	40 (36.4)
Wanted no more	29 (4.1)	21 (0.79)	5 (4.5)
Don’t know	0 (0)	6 (0.23)	0 (0)
No response	0 (0)	11 (0.42)	16 (14.5)
Unmet need for modern contraception	*N* = 1044	*N* = 4180	*N* = 181
No unmet need	845 (79.5)	3264 (78.1)	123 (68.0)
Unmet need for spacing	184 (19.4)	890 (21.3)	55 (30.4)
Unmet need for limiting	15 (1.2)	26 (0.62)	3 (1.7)
Total unmet need	199 (20.5)	916 (21.9)	58 (32.0)

^a^ Numbers and percentages may not match exactly because the analysis used sampling weights to account for the sampling design

Unmet need for modern contraception was 20.5%, made up almost entirely of unmet need for spacing (Table 2).

In Nasarawa, 90.7% of married women aged 15 to 19 years had been sexually active during the previous 12 months. The mean age of first sexual intercourse was 15.1 years (SD 1.7). A total of 3913 (81.3%) married women surveyed had ever been pregnant (Table 2). Overall, 55.0% (2650 of 4816) of married women surveyed had given birth. The mean age at first birth was 16.3 years (SD 1.6). Overall, 13.1% of births were reported as mistimed. Unmet need for modern contraception was 21.9%, made up almost entirely of unmet need for spacing (Table 2).

In Mwanza, 93.1% of married women aged 15 to 19 years had been sexually active during the previous 12 months. The mean age of first sexual intercourse was 16.0 years (SD 1.6). A total of 167 (83.1%) married women surveyed had ever been pregnant (Table 2). Overall, 54.7% (110 of 201) of married women surveyed had given birth. The mean age at first birth was 17.0 years (SD 1.2). Overall, 36.4% of births were reported as mistimed. Unmet need for modern contraception was 32.0%, made up almost entirely of unmet need for spacing (Table 2).

### Family planning characteristics of married girls aged 15 to 19 years

#### Oromia

mCPR for married women aged 15 to 19 years was 47.2%. Injectables were the most commonly used modern method (35.9%), followed by implants (7.9%) (Table 3). Traditional methods were used by 0.26% of respondents. Of those using a modern method of contraception, 93.4% were using injectables or long-acting methods. Use of contraception was not associated with the number of children a woman had (Table 4).

**Table 3:** Contraception use by married women aged 15–19 years

Characteristic	%, (95% CI) ^a^		
	Oromia, Ethiopia *N* = 1198	Nasarawa, Nigeria *N* = 4816	Mwanza, Tanzania *N* = 201
Any method	47.5 (37.3–57.8)	10.6 (9.6–11.6)	20.4 (13.9–28.9)
Any modern method ^b^	47.2 (37.0–57.7)	8.7 (7.9–9.6)	19.4 (13.4–27.3)
Modern method			
Implant	7.9 (5.2–11.9)	1.8 (1.5–2.3)	7.5 (4.3–12.7)
IUCD	0.29 (0.10–0.86)	0.10 (0.04–0.29)	1.5 (0.43–5.0)
Injectables	35.9 (27.9–44.8)	2.4 (2.0–3.0)	4.5 (2.4–8.3)
Oral contraceptive pill	2.3 (1.2–4.5)	1.1 (0.86–1.5)	0.50 (0.07–3.6)
Emergency pill	0.17 (0.05–0.59)	0.44 (0.28–0.67)	0
Male condom	0	2.1 (1.7–2.6)	1.5 (0.45–4.8)
Standard Days Method	0.46 (0.17–1.2)	0.12 (0.06–0.28)	3.0 (0.86–9.9)
Other modern method	0.12 (0.02–0.88)	0.56 (0.38–0.82)	1.0 (0.23–4.2)
Any traditional method	0.26 (0.08–0.89)	1.9 (1.5–2.4)	1.0 (0.22–4.4)
Not currently using	52.5 (42.2–62.7)	89.4 (88.3–90.3)	79.6 (71.2–86.1)
Don’t know	0	0.08 (0.03–0.22)	0

^a^ Numbers and percentages may not match exactly because the analysis used sampling weights to account for the sampling design

^b^ Modern methods include male and female sterilisation, contraceptive implants, intrauterine contraceptive devices (IUCD), injectables, oral contraceptive pill, emergency contraceptive pill, male condom, female condom, Standard Days Method (SDM), Lactational Amenorrhoea Method (LAM), diaphragm, spermicides, foams and jelly

**Table 4:** Family planning characteristics of married women aged 15–19 years

Characteristic	n (%) ^a^		
	Oromia, Ethiopia *N* = 1198	Nasarawa, Nigeria *N* = 4816	Mwanza, Tanzania *N* = 201
Have you seen or heard about contraception in past 12 months			
Yes	1085 (89.1)	1464 (30.4)	181 (90.1)
No	90 (8.3)	3333 (69.2)	20 (9.9)
Don’t know	23 (2.6)	11 (0.23)	0 (0)
No response	0 (0)	8 (0.17)	0 (0)
Contraception information source in past 12 months ^b^			
Radio	211 (20.2)	350 (23.9)	51 (28.2)
Television	76 (6.1)	173 (11.8)	17 (9.4)
Hospital/health centre/clinic	229 (20.4)	808 (55.2)	106 (58.6)
HEW/CHW	324 (31.8)	27 (1.8)	4 (2.0)
Pharmacy/chemist	5 (0.52)	115 (7.9)	38 (21.0)
Teachers	193 (17.4)	5 (0.34)	6 (3.3)
Friends/peers	265 (23.6)	408 (27.9)	41 (22.7)
Neighbours	250 (23.6)	302 (20.6)	60 (33.2)
Spouse/partner	87 (7.1)	73 (5.0)	1 (0.55)
Parent/guardian	103 (8.8)	43 (2.9)	18 (9.9)
Agreed with misconception about contraception			
Some modern contraception can stop an adolescent woman from ever being pregnant again even after she stops using it	298 (26.7)	1096 (48.2)	90 (44.8)
If a modern contraception changes an adolescent woman’s menstrual bleeding, it’s bad for her health and can harm her womb	590 (53.3)	1207 (53.1)	110 (54.7)
Some modern contraceptives can make adolescent women permanently fat	519 (46.6)	1387 (61.1)	109 (54.2)
Adolescent women who use modern contraception are promiscuous	124 (11.5)	952 (41.9)	100 (49.8)
Agreed with benefits about contraception			
Preventing unwanted pregnancies is a benefit of contraception	1000 (90.7)	2058 (90.6)	169 (84.1)
Some contraception methods reduce sexually transmitted infections	371 (34.1)	1696 (74.7)	69 (34.3)
Modern contraception can help an adolescent woman delay the birth of her first child, if she wants to	963 (85.8)	1963 (86.4)	175 (87.1)
After she begins to have children, modern contraception can allow an adolescent woman to decide when to have another child	951 (84.1)	2028 (89.3)	175 (87.1)
Using modern contraception can allow an adolescent woman girl to complete her education, find a better job and have a better life	965 (86.1)	2065 (90.9)	165 (82.1)
Married adolescent women’s approval of married couples using a modern contraceptive method to avoid or delay pregnancy			
Yes	1020 (90.8)	1778 (78.3)	158 (78.6)
No	58 (6.5)	437 (19.2)	39 (19.4)
Don’t know	20 (2.5)	56 (2.5)	4 (2.0)
No response	1 (0.15)	1 (0.04)	0 (0)
Husbands’ approval of married couples using a modern contraceptive method to avoid or delay pregnancy	*N* = 136	*N* = 326	*N* = 16
Yes	127 (91.0)	155 (66.8)	15 (93.8)
No	9 (9.0)	75 (32.3)	1 (6.2)
Don’t know	0 (0)	2 (0.86)	0 (0)
Contraceptive use by no. of children	n/N	n/N	n/N
No children	264/600 (39.9)	102/2328 (4.4)	4/97 (4.1)
1 child	326/520 (56.8)	270/1665 (16.2)	31/85 (36.5)
2 or more children	39/78 (43.2)	137/823 (16.7)	6/19 (31.6)

^a^ Numbers and percentages may not match exactly because the analysis used sampling weights to account for the sampling design

^b^ Respondents were able to state multiple sources of information on contraception

Most married women had seen or heard of contraception in the past 12 months (89.1%). Health extension workers were the most common source of information on contraception (31.8%). The majority of married women aged 15 to 19 years knew the benefits of modern contraception. However, many respondents also had misconceptions about modern contraception (Table 4).

**Fig. 1:**
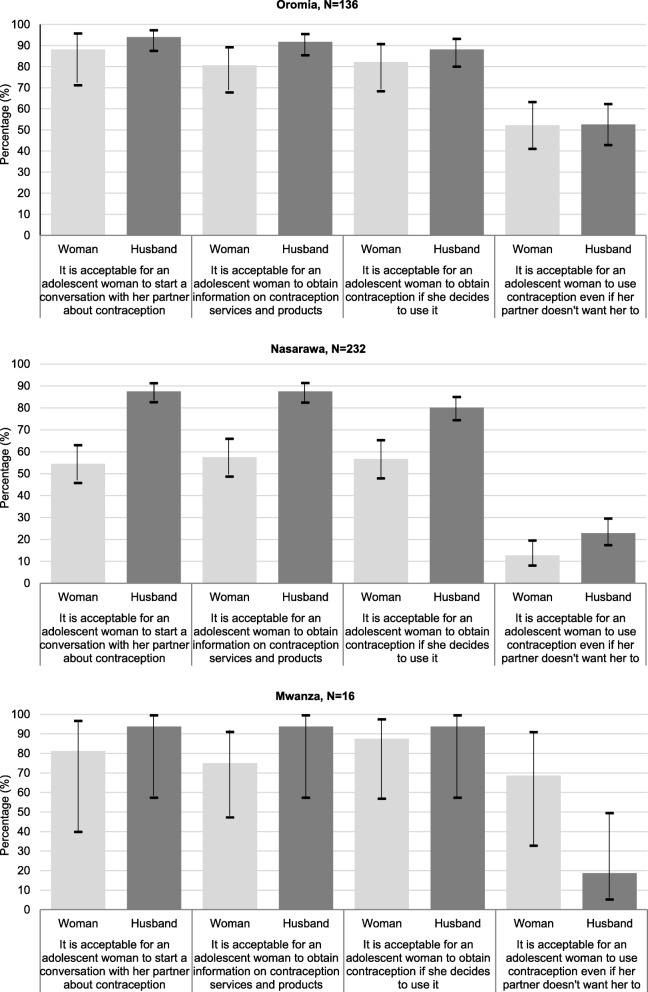
Attitudes towards self-efficacy of adolescent women to access contraception (%, 95 Cl)

Overall, attitudes of female respondents and their husbands towards contraceptive use were positive and broadly similar (Table 4). Figure 1 compares the attitudes of a subgroup of married women and their husbands towards self-efficacy of adolescent women to access and use contraceptive methods. The majority of married women and their husbands said it was acceptable for an adolescent woman to start a conversation with her partner about contraception, obtain information on contraception services and products, and that it was acceptable for an adolescent woman to obtain contraception if she needs it. However, only about half of married women and their husbands said it was acceptable for an adolescent woman to use contraception even if her partner does not want her to (Fig. 1).

#### Nasarawa

mCPR for married women aged 15 to 19 years was 8.7%. Injectables were the most common modern method (2.4%), followed by the male condom (2.1%) and implants (1.8%) (Table 3). Traditional methods were used by 1.9% of respondents. Of those using a modern method of contraception, 49.4% were using injectables or long-acting methods. The percentage of married women using contraception was higher in those who had at least one child (Table 4).

About a third of married women had seen or heard of contraception in the past 12 months (30.4%). Health facilities, including hospitals and health centres, were the most common source of information on contraception (55.2%) (Table 4). The majority of married women aged 15 to 19 years knew the benefits of modern contraception. However, many respondents also had misconceptions about modern contraception (Table 4).

Overall, the majority of female respondents (78.3%) and husbands (66.8%) in Nasarawa approved of married couples using a modern contraceptive method to avoid or delay pregnancy (Table 4). Few married women (12.7%) and their husbands (22.8%) said it was acceptable for an adolescent woman to use contraception even if her partner does not want her to (Fig. 1). However, a higher percentage of husbands said it was acceptable for an adolescent woman to access family planning information and contraceptive products, and start a conversation with her partner about contraception, compared with the percentage of married women (Fig. 1).

#### Mwanza

mCPR for married women aged 15 to 19 years was 19.4%. Implants were the most commonly used modern method (7.5%), followed by injectables (4.5%) (Table 3). Traditional methods were used by 1.0% of respondents. Of those using a modern method of contraception 69.6% were using injectables or long-acting methods. The percentage of married women using contraception was higher in those who had at least one child (Table 4).

Most married women had seen or heard of contraception in the past 12 months (90.1%). Health facilities, including hospitals and health centres, were the most common source of information (58.6%). The majority of married women aged 15 to 19 years knew the benefits of modern contraception. However, many respondents also had misconceptions about modern contraception (Table 4).

Overall, the majority of female respondents (78.6%) and husbands (93.8%) in Mwanza approved of married couples using a modern contraceptive method to avoid or delay pregnancy (Table 4). Attitudes of married women and husbands are broadly similar with respect to an adolescent woman being able to obtain information on contraception services and products, being able to obtain contraception if she needs it, and being able to start a conversation with her partner about contraception. However, fewer husbands (18.8%) said it was acceptable for an adolescent woman to use contraception even if her partner does not want her to, compared to what married women (68.8%) said they felt able to do (Fig. 1).

## Discussion

There was significant variation in modern contraceptive prevalence for married women aged 15 to 19 years between the three study settings. The high use of injectables and implants among those using contraception is an encouraging finding. Our findings on contraceptive use were more positive than results from the most recent country-specific DHS [14–16]. In the most recent DHS, current use of modern contraception among married women aged 15 to 19 years was 31.8, 1.2 and 13.3% in Ethiopia, Nigeria and Tanzania, respectively. However, mCPR varies significantly by region, and by rural compared to urban areas in the three countries [14–16]. Differences also could be partly explained by upward secular trends in voluntary contraceptive use between the most recent DHS survey and mid-2017 when we conducted our baseline surveys. Our findings are consistent with previous studies focusing on sexually active women aged 15 to 19 years showing low contraceptive use in this subgroup of women, considerably variation by geographic region of sub-Saharan Africa, and Ethiopia showing significant progress in recent years regarding use of modern contraceptives among sexually active adolescents [19–22]. In Ethiopia, a number of large-scale initiatives have recently been launched, including a national strategy and campaign to tackle child marriage [23], and expansion of the Ethiopian Health Extension Programme, which aims to provide counselling and family planning to women of all ages, and addresses community misconceptions surrounding modern contraception [24–26]. Such initiatives could, in part, explain the higher mCPR in Oromia compared to Nasarawa and Mwanza.

Our findings suggest that unmet need for modern contraception is high for married women aged 15 to 19 years across the three settings. Unmet need for modern contraception was made up almost entirely of unmet need for spacing. Our findings were consistent with results from the most recent country-specific DHS [14–16], and previous community-based cross-sectional studies [27–29] showing variation by geographic region of sub-Saharan Africa, and that unmet need for modern contraception is mostly for spacing in this subgroup of women. Although mCPR was greater in Oromia than in Nasarawa, we found that unmet need for modern contraception was similar across the two settings. The proportion of married women who wanted to delay or limit their next pregnancy, but who were not using contraception, was similar in both settings. Nasarawa had a much higher proportion of married women who wanted to become pregnant within the next 2 years and consequently were not using contraception. These findings highlight the fact that fertility intentions differ between the settings. While addressing unmet need is important, higher levels of contraceptive use may only be achieved in Nasarawa if there is a population-level shift in attitudes towards fertility and ideal family size. Married women aged 15 to 19 years in Mwanza had a higher unmet need than those in either Oromia or Nasarawa. This can be explained by the larger proportion of pregnant women in Mwanza who reported their current pregnancy as mistimed and by the larger proportion of fecund, not pregnant married women not currently using contraception who wanted to delay the birth of another child. Socio-cultural and structural barriers might be contributing to the gap between adolescent women’s reproductive intentions and their use of modern contraception [11]. The findings suggest that these barriers might be greater for married women aged 15 to 19 years in Mwanza compared to Oromia and Nasarawa.

The cross-settings comparison also revealed some differences in other family planning characteristics. Married women in Nasarawa and Mwanza more commonly accessed contraception information from health facilities compared to those in Oromia who mostly obtained this information from health extension workers and community health workers. This may reflect the difference in healthcare seeking behaviours or access to contraception information for married women aged 15 to 19 years in Oromia, Nasarawa and Mwanza, which is likely to be determined, in part, by the social and policy context of the three study settings, including the success of the Ethiopian Health Extension Program [24–26]. Despite different sources of information, married women across the three settings had high levels of knowledge about the benefits of contraception and positive attitudes to married couples using modern contraception, albeit some misconceptions and low mCPR, especially in Nasarawa.

Overall, husbands in Oromia, Nasarawa and Mwanza had broadly similar positive attitudes to modern contraception compared to their adolescent wives with respect to approving of married couples using a modern contraceptive method to avoid or delay pregnancy. In Nasarawa, husbands tended to be older than in Ethiopia and Mwanza, and generally had more positive attitudes towards the self-efficacy of married women aged 15 to 19 years to access and use modern contraception compared to their adolescent wives. Given the low mCPR in Nigeria, it is difficult to interpret this finding; it might reflect selection, acquiescence or social desirability bias in this sub-group of respondents. If we are to take these results at face value, they may highlight the need for a planned focus on partner communication for married women and/or couples-counselling to help create a supportive environment for accessing and using modern contraception in this setting.

A strength of our study is that we were able to compare population-based data from three settings. In addition, the large sample of married women aged 15 to 19 years and the large variety of indicators for which data were collected to describe sexuality and fertility, including from their husband’s perspective, allowed us to present an in-depth analysis of the characteristics of married women aged 15 to 19 years, and their husbands. An important limitation is that our study settings were selected for the purposes of baseline surveys for an outcome evaluation of the A360 programme, and therefore not necessarily selected to be nationally or sub-nationally representative, thus limiting the ability to generalise our findings to the wider population of married women aged 15 to 19 years and their husbands in the three countries. This study relied solely on quantitative data, which limits our ability to understand the effects of sociocultural factors on patterns in sexuality and fertility characteristics in married women aged 15 to 19 years in these specific settings. Such factors may be better explored through qualitative studies. We did not collect data on abortion, which may also be an important dimension of adolescent’s sexuality and fertility.

Context-specific attitudes towards fertility and ideal family size, and socio-cultural and structural barriers need to be taken into account when designing adolescent sexual and reproductive health programmes. Across all three countries, adolescents who have a high school level education or above, who are in urban areas, and who are in the highest wealth quintiles use significantly more modern contraception as compared to their peers who have primary-level education, live in rural areas, or who belong to the lowest wealth quintiles [19]. Differences across the three countries in terms of policies, strategies, and investment by governments in women’s, children’s, and adolescents’ health, will determine the extent to which keeping adolescent girls in school, curtailing child marriage, and access to modern contraception for all women of reproductive age will impact on the success of adolescent sexual and reproductive health programmes. Married women aged 15 to 19 years across the three settings have high levels of knowledge about benefits of contraception, but misconceptions are widespread and mCPR is low, especially in Nasarawa. This highlights a need to build trust and credibility of contraceptive products among married women aged 15 to 19 years, by addressing fears, misconceptions and myths, and working with communities to help tackle prevailing social norms and create a supportive environment for accessing services. Finally, of those using modern contraception, fewer married women in Nasarawa and Mwanza were using injectables and implants compared to Oromia. Effective family planning counselling in these settings should focus on shifting the method mix towards injectables and long-acting methods for birth spacing and, when discussing method choice, must prepare women for the possibility that they will experience side effects and provide them with the information and tools to overcome them.

## Conclusions

Globally, patterns of contraceptive use among adolescents are heterogeneous across countries and across regions and sociodemographic subgroups within countries [19, 30]. Because of this, no single intervention or strategy will suit all situations [7, 31]. This study highlights the importance of describing variations and differences in modern contraceptive use and unmet need among married adolescent women in order to better address their needs. The higher use of modern contraception in Ethiopia is an indication that, when proper policies and investments are made (e.g. the Health Extension Workers Programme), it is possible to have considerable impact in a short period of time. Further research should include systematic analyses of the reasons for success of the policies and strategies being implemented in Ethiopia. It is critical for evidence-based policy making and programme design to identify the components of these policies and strategies which can be implemented or adapted for success in other contexts, and share these findings with other countries. Improving the quality, analysis, and utilisation of data is key to understanding where and which young people have been reached, where gaps remain, and how to bring effective programmes to scale. We acknowledge that our findings cannot be generalised to the national level. Nonetheless, we believe our findings have some interesting implications for programmers and policy-makers.

## Abbreviations

A360: Adolescents 360
CI: Confidence interval
DHS: Demographic health surveys
EA: Enumeration area
FP2020: Family planning 2020
HCD: Human-centred design
IQR: Interquartile range
IUCD: Intrauterine contraceptive devices
LAM: Lactational amenorrhoea method
LGA: Local government area
mCPR: Modern contraceptive prevalence rate
PSU: Primary sampling unit
SD: Standard deviation
SDGs: Sustainable development goals
SDM: Standard days method
SRH: Sexual and reproductive health
UN: United Nations
